# Functional recovery after dantrolene-supplementation of cold stored hearts using an ex vivo isolated working rat heart model

**DOI:** 10.1371/journal.pone.0205850

**Published:** 2018-10-12

**Authors:** Jeanette E. Villanueva, Ling Gao, Hong C. Chew, Mark Hicks, Aoife Doyle, Min Ru Qui, Kumud K. Dhital, Peter S. Macdonald, Andrew Jabbour

**Affiliations:** 1 Cardiac Physiology and Transplantation, Victor Chang Cardiac Research Institute, Darlinghurst, NSW, Australia; 2 University of New South Wales, Randwick, NSW, Australia; 3 Department of Clinical Pharmacology and Toxicology, St Vincent’s Hospital, Darlinghurst, NSW, Australia; 4 Department of Anatomical Pathology, SydPath, St Vincent’s Hospital, Darlinghurst, NSW, Australia; 5 Heart and Lung Transplant Unit, St Vincent’s Hospital, Darlinghurst, NSW, Australia; Indiana University School of Medicine, UNITED STATES

## Abstract

The ryanodine receptor antagonist dantrolene inhibits calcium release from the sarcoplasmic reticulum and reduces cardiac ischaemia-reperfusion injury (IRI) in global warm ischaemia models however the cardioprotective potential of dantrolene under hypothermic conditions is unknown. This study addresses whether the addition of dantrolene during cardioplegia and hypothermic storage of the donor heart can improve functional recovery and reduce IRI. Using an *ex vivo* isolated working heart model, Wistar rat (3 month and 12 month) hearts were perfused to acquire baseline haemodynamic measurements of aortic flow, coronary flow, cardiac output, pulse pressure and heart rate. Hearts were arrested and stored in Celsior preservation solution supplemented with 0.2–40 μM dantrolene for 6 hours at 4°C, then reperfused (15 min Langendorff, 30 min working mode). In 3-month hearts, supplementation with 1 μM dantrolene significantly improved aortic flow and cardiac output compared to unsupplemented controls however lactate dehydrogenase (LDH) release and contraction bands were comparable. In contrast, 40 μM dantrolene-supplementation yielded poor cardiac recovery, increased post-reperfusion LDH but reduced contraction bands. All 3-month hearts stored in dantrolene displayed significantly reduced cleaved-caspase 3 intensities compared to controls. Analysis of cardioprotective signalling pathways showed no changes in AMPKα however dantrolene increased STAT3 and ERK1/2 signaling in a manner unrelated to functional recovery and AKT activity was reduced in 1 μM dantrolene-stored hearts. In contrast to 3-month hearts, no significant improvements were observed in the functional recovery of 12-month hearts following prolonged storage in 1 μM dantrolene. *Conclusions*: Dantrolene supplementation at 1 μM during hypothermic heart preservation improved functional recovery of young, but not older (12 month) hearts. Although the molecular mechanisms responsible for dantrolene-mediated cardioprotection are unclear, our studies show no correlation between improved functional recovery and SAFE and RISK pathway activation.

## Introduction

A major goal in heart transplantation is preventing and mitigating ischaemic injury during organ procurement, and cold ischaemia during storage and transportation. The failure of ATP-dependent ion pumps during hypoxia causes detrimental increases in cytosolic Na^+^ and Ca^2+^ concentrations[1]. Warm blood reperfusion and reoxygenation of the donor heart causes further injury due to rapid ATP flux and increased Ca^2+^ release from the sarcoplasmic reticulum (SR) via ryanodine receptors (RyRs). High cytosolic Ca^2+^ concentrations promotes rapid Ca^2+^ oscillations and calcium-induced calcium release contributing towards mitochondrial Ca^2+^ uptake, mitochondrial permeability transition pore (mPTP) opening and subsequent apoptosis and necrosis[2]. Pharmacological modulation of Ca^2+^ release from RyRs is one approach that could reduce ischaemia-reperfusion injury (IRI) of the donor heart caused by Ca^2+^ overload.

The ryanodine receptor antagonist dantrolene is clinically used to treat malignant hyperthermia in individuals with a mutation in the skeletal muscle RyR1 isoform[3, 4]. Dantrolene also binds the cardiac RyR2 isoform[3, 5] and inhibits SR Ca^2+^ release by altering RyR conformation from an ‘unzipped’ conformation under stress conditions (e.g., hypoxia) to a ‘zipped’ conformational state that retains calmodulin binding and prevents diastolic Ca^2+^ release[6–8]. Several studies have shown the anti-arrhythmogenic effects of dantrolene. Dantrolene inhibited slow inward currents and prolonged the action potential duration in isolated sinoatrial and atrioventricular nodes [9] and reduced arrhythmias in a murine model of exercise-induced ventricular tachycardia that contain a RyR2 mutation analogous to the human catecholaminergic polymorphic ventricular tachycardia (CPVT)[10]. Spontaneous Ca^2+^ release events from SR RyRs were significantly reduced in dantrolene-treated CPVT myocytes and heart failure-derived cardiomyocytes [11–13]. Also, dantrolene administration during cardiopulmonary resuscitation aided the return of spontaneous circulation after defibrillation in a porcine VF model[14]. In the same study, dantrolene infusion of Langendorff-perfused rabbit hearts increased resistance to isoproterenol-induced VF and reduced diastolic Ca^2+^ release from RyR2[14]. These studies show that dantrolene treatment can correct abnormal cardiac physiological events caused by altered Ca^2+^ release.

Previous studies have used various myocardial ischaemia models to test whether dantrolene can reduce IRI [15–18]. Administration of dantrolene to Langendorff-perfused hearts prior to global normothermic ischaemia significantly improved left ventricular developed pressure compared to vehicle controls[16] and dantrolene administration at reperfusion elicited a dose-dependent protective effect indicated by reduced LDH release and tissue necrosis[18]. Despite biochemical indicators demonstrating reduced IRI, dantrolene doses exceeding 16 μM exerted a negative inotropic effect[18]. In a similar study, dantrolene administration (25 μM or 100 μM) during the initial 15 minute reperfusion reduced creatine kinase release despite a lack of functional recovery across all treatment groups[17]. In an isoproterenol-induced myocardial ischemia model, dantrolene administration reduced cardiac troponin levels, an indicator of myocardial cellular injury, and reduced the severity of histological cardiac damage indicated by eosinophilia and interstitial oedema[15]. These studies provide evidence supporting a protective role for dantrolene in experimental warm ischaemia models. However, to our knowledge, studies testing the cardioprotective efficacy of dantrolene during prolonged cold ischaemia in the context of donor hearts have not been performed. The present study uses an *ex vivo* rodent working heart reperfusion model to test whether dantrolene supplementation of Celsior cardiac arresting and preservation solution during 6 hour hypothermic storage is beneficial for: i) cardiac functional recovery; ii) necrosis and apoptotic indices; iii) pro-survival signalling kinases ERK, AKT, STAT3, and AMPKα. Identifying novel agents that further reduce IRI and improve cardiac recovery of donor hearts after prolonged cold ischaemia has the potential to increase donor heart availability and alleviate heart transplant wait lists.

## Materials and methods

### Animals

Male Wistar rats aged approximately three-months (325-440g) and twelve-months (650-940g) were obtained from the Animal Resources Centre (Canning Vale, WA, Australia) and Charles River Laboratories (Kingston, NY, USA) respectively. Animals received humane care in compliance with the National Health and Medical Research Council (Australia) guidelines. All procedures were approved by the Animal Ethics Committee of the Garvan Institute of Medical Research (Sydney, Australia). Animal Research Authority Ref #12/28, #15/28, and #15/05.

### Ex vivo perfusion model

The isolated working rat heart model used has been previously described[19–22]. Rats were anesthetized with an intraperitoneal injection of ketamine (80mg/kg; Cenvet Australia, Kings Park, NSW, Australia) and xylazine (10mg/kg; Provet, Eastern Creek NSW, Australia). Prior to heart excision, 150 IU heparin (Pfizer, West Ryde, NSW, Australia) was administered via the renal vein. Once excised, the heart was cannulated and immediately perfused retrogradely on a Langendorff perfusion apparatus with Krebs-Henseleit buffer (KHB) for 10 mins at 37°C (composition (mM): NaCl 118; KCl 4.7; MgSO_4_ 1.2; KH_2_PO_4_ 1.2; NaHCO_3_ 25; CaCl_2_ 1.4; glucose 11; pH 7.3–7.4) at a hydrostatic pressure of 100 cm H_2_O. After stabilization, perfusion was switched to working mode and perfused via a left atrial cannula at a hydrostatic pressure of 15 cm H_2_O. The working heart ejected perfusate into the aortic cannula against a fixed pressure (100 cm H_2_0) for 15 min, and functional parameters of aortic pressure, aortic flow (AF), coronary flow (CF), cardiac output (CO), heart rate (HR), and pulse pressure (PP) continuously measured. Hearts with a baseline AF <35 ml/min, HR <200 bpm, or CF <10 ml/min were excluded. Coronary effluent was collected at the end of baseline measurements.

### Prolonged IRI protocol

Hearts were arrested by infusion of Celsior preservation solution (4°C; Genzyme, Naarden, Netherlands) alone (control) or with dantrolene sodium salt (0.2, 0.4, 1, 4, and 40 μM; Sigma-Aldrich, St. Louis, MO, USA) into the coronary circulation for 3 min from a reservoir 60 cm above the heart. Hearts were removed from the Langendorff apparatus with the cannulae kept *in situ* and stored at 4°C in 100 ml of the designated arresting solution for 6 hours. After storage, hearts were placed on the perfusion apparatus and reperfused in Langendorff mode for 15 min before switching to working mode. Functional parameters were recorded for 30 min during working mode. Cardiac functional recovery at the end of reperfusion was calculated as a percentage of the pre-storage baseline value.

### Assessment of lactate dehydrogenase release

Coronary effluent was collected at baseline, 15 min, 30 min, and 45 min reperfusion time points. Myocardial injury based on lactate dehydrogenase levels was assessed using the In Vitro Toxicology Assay Kit (TOX7; Sigma-Aldrich) according to manufacturer’s instructions. Results are expressed as arbitrary units of colorimetric absorbance (490 nm) following normalisation with coronary flow values[23].

### Western blot analysis

Left ventricular tissue collected after reperfusion was snap frozen and stored at -80°C. Tissue homogenates were processed as previously described[21]. Protein (30 μg) was added to Laemlli sample buffer (BioRad Laboratories, Gladesville, NSW, Australia) then heated at 95°C for 10 min and run on a 4–20% Mini-PROTEAN TGX precast gel (BioRad Laboratories) at 80 V for 15 min then 120 V for 60 min. Proteins were transferred onto an Immobilon-P PVDF 0.45 μm membrane (Merck Millipore, Bayswater, VIC, Australia) at 100 V for 80 min in transfer buffer containing 20% methanol. Membranes were washed in TBS-0.1% Tween (TBS-T) for 5 min, blocked for 1 h at room temperature in 5% (w/v) skim milk+TBS-T, then washed in TBS-0.1% Tween (TBS-T) for 5 min. Membranes were incubated with primary antibody overnight at 4°C in TBS-T. Primary antibodies (1:1000; Cell Signaling, Beverly, MA, USA): phospho-AMPKα (Thr172, 40H9), AMPKα, phospho-p44/42 MAPK (ERK1/2; T202/Y204), p44/42 MAPK, phospho-AKT (S473), AKT, phospho-STAT3 (Tyr705), STAT3 (79D7), phospho-connexin 43 (S368), calmodulin, α-tubulin (1:3000; Sigma-Aldrich). Membranes were incubated with secondary anti-rabbit (1:2000 in 5% skim milk + TBS-T) or anti-mouse (1:5000 in 1% BSA + TBS-T) IgG-horseradish peroxidase antibody (both GE healthcare, Rydalmere, NSW, Australia) for 2 h at room temperature. Protein bands were visualized using SuperSignal West Pico Chemiluminescent (Life Technologies, Scoresby, VIC, Australia), digitized, and quantified using ImageJ (Version 1.48v, National Institutes of Health, USA).

### Histopathology

Myocardial samples were collected after reperfusion and stored in 4% paraformaldehyde (Sigma-Aldrich). Formalin-fixed tissues were paraffin embedded and 5 μm sections were stained with hematoxylin and eosin (H&E). Blinded analysis of contraction bands was performed by an experienced anatomical pathologist (MRQ) and quantified as the average number of contraction bands in four high-powered (40X) fields. Cleaved-caspase 3 (1:300, ab13847, Abcam, Cambridge, MA, USA) staining was quantified using ImageJ (National Institutes of Health, Bethesda, MD, USA) and expressed as the average percentage of cleaved-caspase 3 positive stained tissue calculated from six high-powered (40X) fields for each heart section.

### Statistical analysis

Statistical analysis was performed using GraphPad Prism (Version 7.03; GraphPad Software Inc., La Jolla, CA, USA). For comparison of baseline cardiac haemodynamics before and after dantrolene addition, data was analysed using a Wilcoxon matched-pairs sign rank test. Recovery from baseline data was analysed using Mann-Whitney unpaired t test versus control group. Functional post-reperfusion data and LDH results were analysed using a two-way ANOVA with Dunnett’s multiple comparisons test. Histopathology and densitometry results were analysed using a one-way ANOVA with Holm-Sidak’s multiple comparisons test. A p value <0.05 was considered statistically significant.

## Results

### Effects of dantrolene on baseline cardiac haemodynamics

To test whether dantrolene would affect baseline cardiac function, hearts were perfused with KHB for 10 min in Langendorff mode to stabilise the heart and then perfused with KHB for 15 min in working mode. Compared to KHB alone, the addition of 4 μM dantrolene to the perfusate during working mode did not alter baseline AF, CF, CO and PP (Table 1). A small increase in HR was observed following the addition of 4 μM dantrolene however this was not statistically significant. Overall 4 μM dantrolene administered during pre-storage perfusion does not alter baseline cardiac haemodynamics.

**Table 1:** Effect of dantrolene on baseline cardiac haemodynamics (mean ± SD).

Functional parameter	KHB only (n = 4)	KHB + 4μM dantrolene (n = 4)	P value*
Aortic flow (ml/min)	42.73 ± 6.41	45.36 ± 3.55	0.625
Coronary flow (ml/min)	20.75 ± 4.03	23.25 ± 4.65	0.125
Cardiac Output (ml/min)	63.48 ± 8.22	68.61 ± 7.01	0.250
Heart rate (bpm)	244.26 ± 41.33	274.43 ± 15.78	0.375
Pulse Pressure (mmHg)	50.06 ± 17.04	43.12 ± 16.92	0.875

KHB, Krebs-Henseleit Buffer.

* *p* values were calculated using the Wilcoxon matched-pairs signed rank test.

### Effects of dantrolene-supplementation during cold storage on donor heart functional recovery

**Fig 1:**
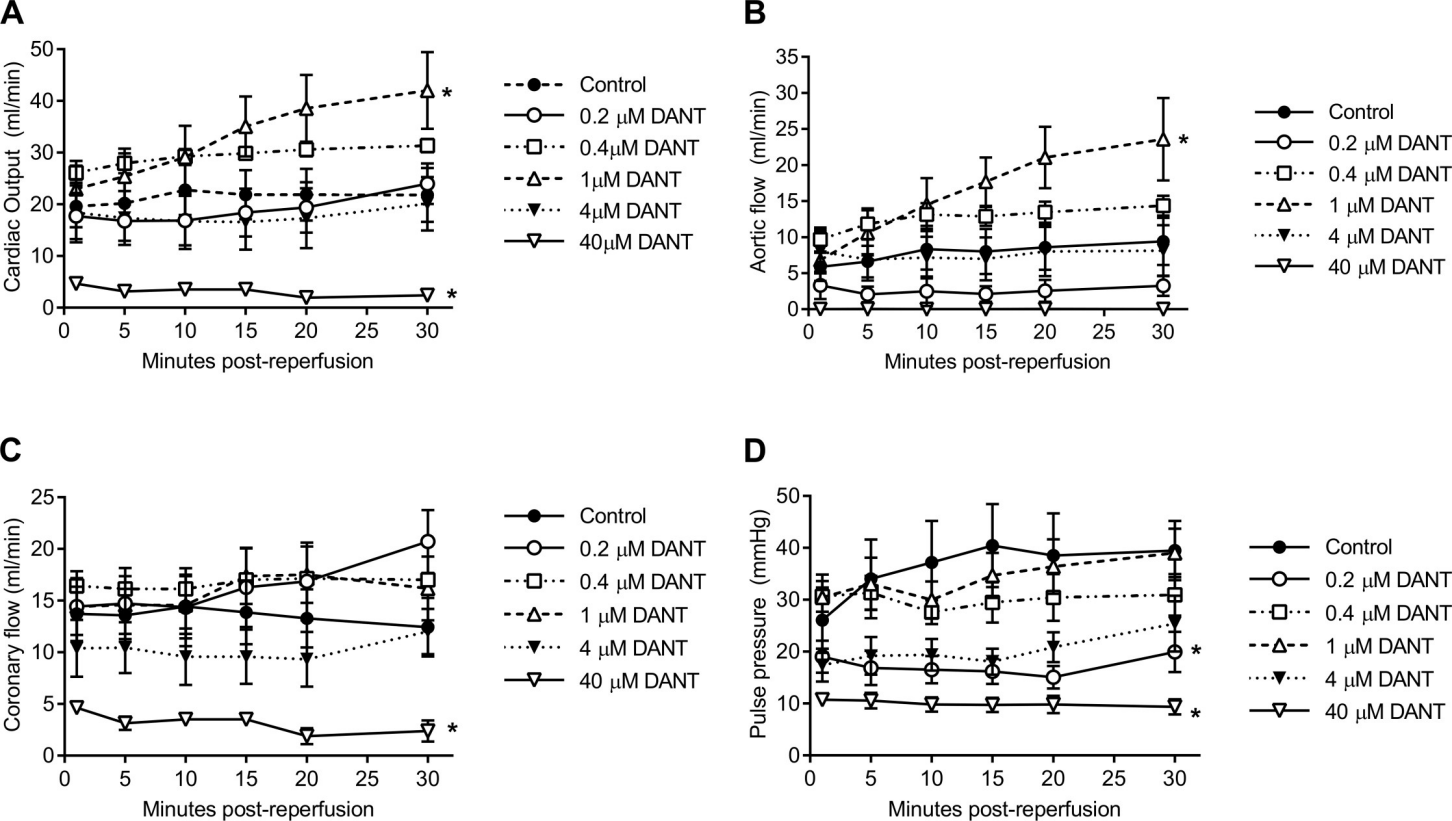
Cardiac functional recovery during 30 minutes working mode reperfusion after dantrolene-supplementation during prolonged cold storage. (A) Cardiac output, ml/min; (B) aortic flow, ml/min; (C) coronary flow, ml/min; (D) pulse pressure, mmHg. Data shown as mean ± SEM. * p<0.05 versus control, two-way ANOVA with Dunnett’s multiple comparisons test, n = 4–8 per group.

**Fig 2:**
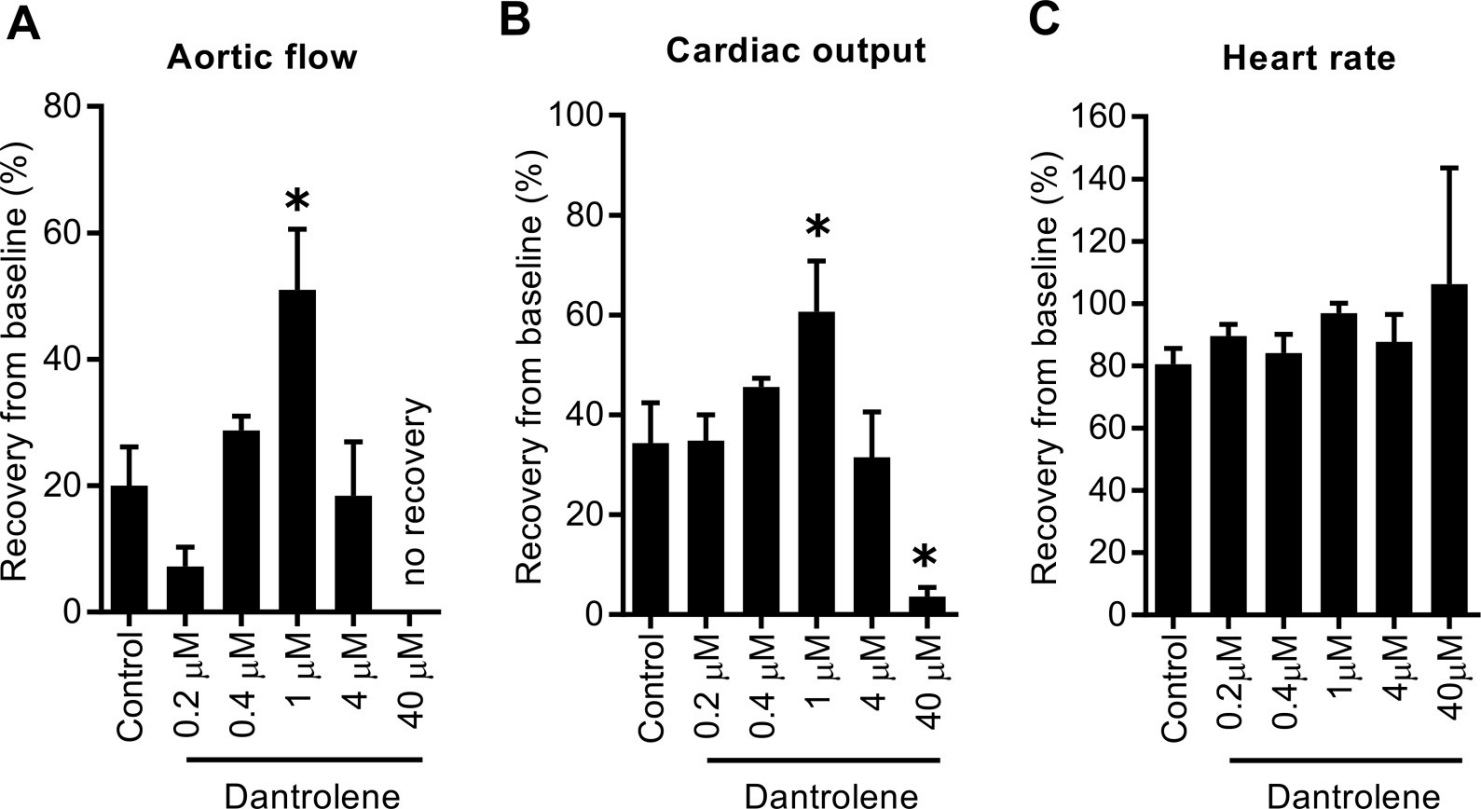
Cardiac functional recovery at the end of 30 minutes working mode reperfusion as a percentage of baseline value. (A) aortic flow; (B) cardiac output, (C) heart rate. Bars represent mean ± SEM; * p < 0.05 versus control, Mann-Whitney unpaired t test; n = 4–8 per group.

To test whether dantrolene-supplementation would affect the cardiac recovery after prolonged cold storage of the donor heart, Celsior preservation solution was supplemented with either 0.2 μM, 0.4 μM, 1 μM, 4 μM, or 40 μM dantrolene. Of the concentrations tested, supplementation with 1 μM dantrolene significantly increased CO during the 30 minute working heart reperfusion compared to unsupplemented control (Fig 1A). This observation was primarily due to a significant increase in AF as CF was only slightly improved with 1 μM dantrolene supplementation (Fig 1B and 1C). In contrast, hearts supplemented with 40 μM dantrolene showed significantly worse AF, CF and CO recovery throughout the 30 minute reperfusion period (Fig 1A–1C). A significant impairment in PP was evident when hearts were stored at the lower dose of 0.2 μM or the higher dose of 40 μM dantrolene compared to unsupplemented controls (Fig 1D). Similar effects of dantrolene-supplementation on cardiac recovery were also observed when functional recovery was analysed as a percentage of its corresponding baseline haemodynamic value, with 1μM dantrolene-supplemented hearts showing significantly improved AF and CO whereas hearts supplemented with 40 μM dantrolene showed the poorest AF and CO recovery (Fig 2A and 2B). There were no differences in HR recovery between unsupplemented controls and dantrolene-supplemented groups (Fig 2C). These data indicate that of the concentrations tested in the current study, supplementation of hearts with 1 μM dantrolene yielded the greatest improvement in cardiac functional recovery after prolonged cold storage.

### Effects of dantrolene-supplementation during cold storage on donor heart cellular injury

**Fig 3:**
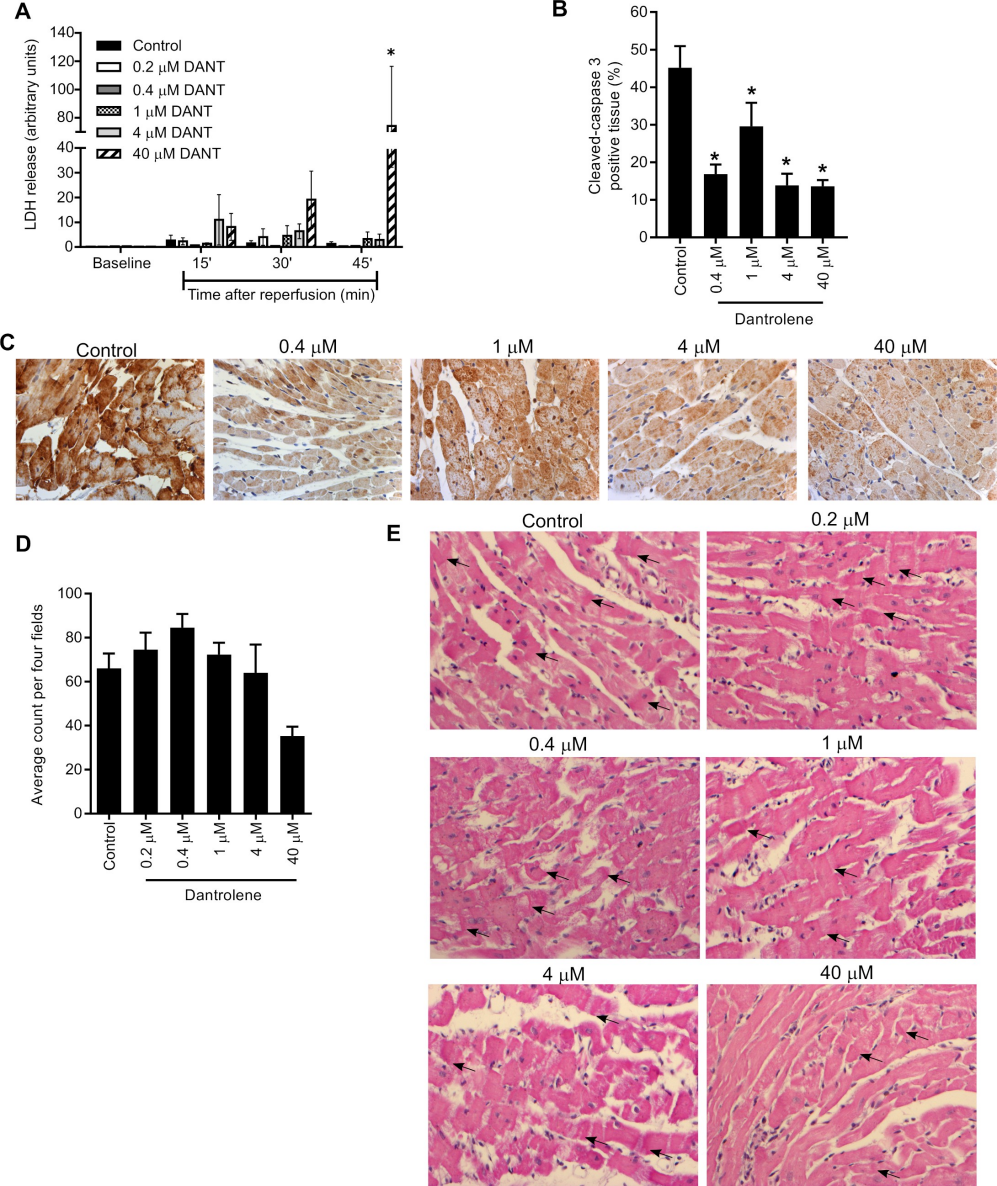
Analysis of cardiac cellular injury post-reperfusion after 6h storage in Celsior alone (control) or dantrolene-supplemented. (A) Lactate dehydrogenase release in coronary effluent collected at baseline and during reperfusion. Bars represent mean ± SEM; n = 4–7 per group; * p<0.05 compared to control at 45 min reperfusion, Dunnett’s multiple comparisons test. (B) Cumulative cleaved-caspase 3 analysis presented as the percentage of cleaved-caspase 3 positive tissue per six high-powered (40X fields). Bars represent mean ± SEM; n = 4 per group *p< 0.05 versus control, Mann-Whitney unpaired t test. (C) Representative cleaved-caspase 3 staining of hearts stored in Celsior alone (control) or supplemented with 0.4–40 μM dantrolene. (D) Contraction band quantification in H&E sections as the average count per four high powered (40X) fields. Bars represent mean ± SEM; n = 4–7 per group; (E) Representative H&E staining depicting contraction bands (black arrows) of hearts stored in Celsior alone (control) or supplemented with 0.2–40 μM dantrolene.

To assess cellular injury of the donor hearts following cold storage, LDH levels were measured in the coronary effluent collected during reperfusion. Compared to unsupplemented controls, LDH release from hearts supplemented with 0.2–4 μM dantrolene were comparable, however, supplementation with 40 μM dantrolene caused a significant increase in LDH release by 45 min reperfusion (Fig 3A). To assess apoptosis at the end of the reperfusion, tissue sections were stained for cleaved-caspase 3 (Fig 3B). Compared to unsupplemented controls, all dantrolene-stored hearts showed a significant reduction in cleaved-caspase 3 positive stained tissue (Fig 3C). To further assess cellular injury in dantrolene-supplemented hearts, tissue sections were H&E stained and examined for the presence of contraction bands as a marker of necrosis. Histological analysis showed that dantrolene did not alter the number of contraction bands evident after reperfusion of cold-stored hearts except when stored in 40 μM dantrolene which caused a 30% reduction in contraction bands (Fig 3D and 3E) but this did not reach statistical significance (ANOVA, p = 0.089). Despite improved recovery of 1 μM dantrolene-supplemented hearts, this was not due to reduced contraction band necrosis or reduced apoptosis since no distinction in LDH release, contraction bands, or cleaved-caspase 3 expression was evident between the different dantrolene concentration groups.

### Cardioprotective-signaling in dantrolene-supplemented hearts

**Fig 4:**
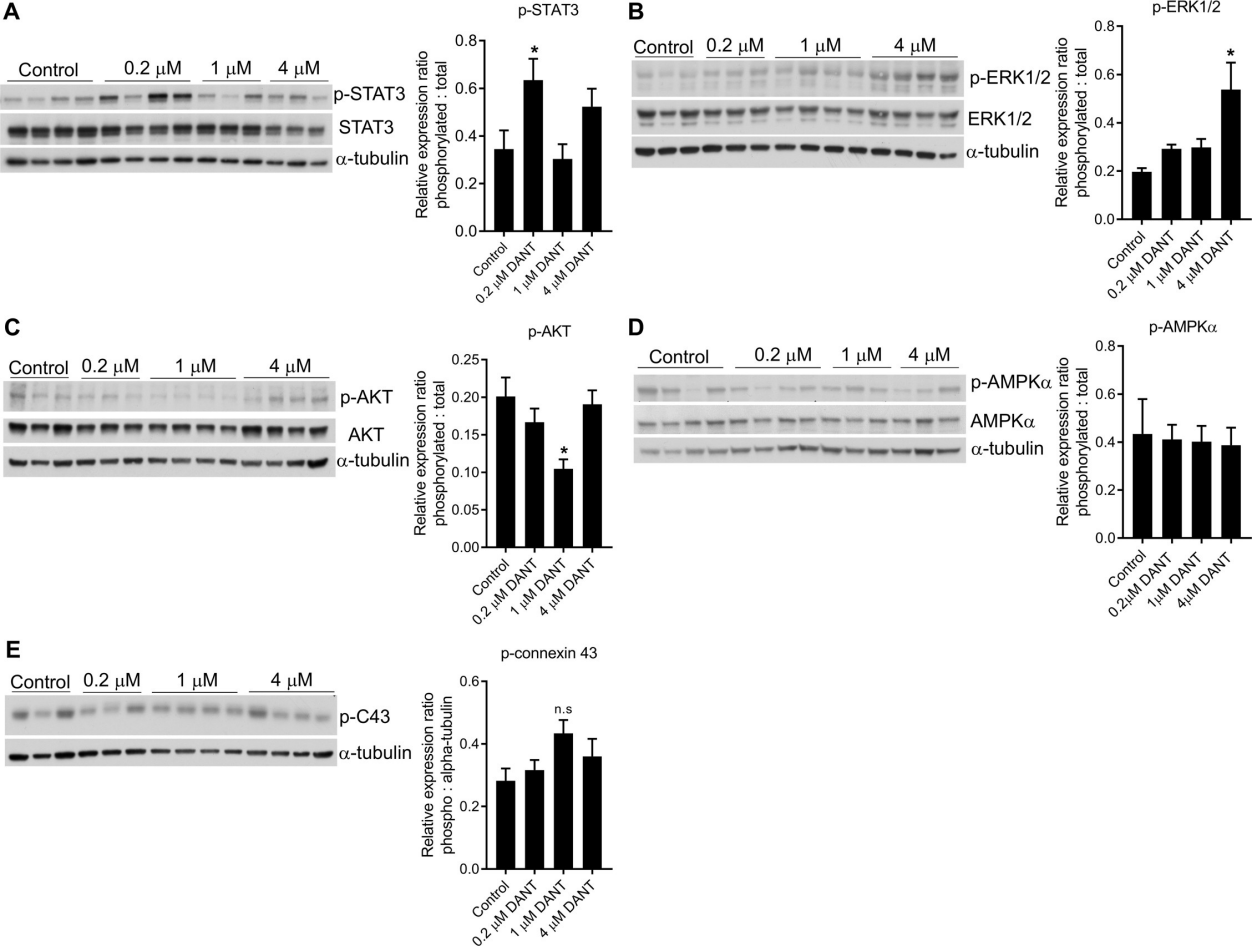
Cardioprotective signalling pathways in dantrolene-stored hearts. Representative western blots and cumulative densitometry analysis of (A) STAT3, (B) ERK1/2, (C) AKT, (D) AMPKα, and (E) connexin 43 (S368). Representative blots of 3–4 biological replicates are shown. Densitometry was calculated from n = 7 biological replicates for each group.

To elucidate the mechanism by which dantrolene was improving cardiac recovery, four signalling pathways implicated in mediating cardioprotection were examined: i) the survivor activating factor enhancement (SAFE) pathway indicated by STAT3; ii)the reperfusion injury survival kinase (RISK) pathway indicated by ERK1/2 and AKT; iii) AMPKα activation; and iv) connexin 43 phosphorylation. Compared to control hearts, a significant increase in STAT3 phosphorylation was observed only in hearts supplemented with 0.2 μM dantrolene (Fig 4A). An increase in ERK1/2 phosphorylation was observed in hearts supplemented with 4 μM dantrolene but not at lower dantrolene concentrations tested (Fig 4B). Contrary to increased RISK signalling with respect to ERK1/2 phosphorylation, AKT phosphorylation was reduced in hearts stored with 1 μM dantrolene (Fig 4C). No significant changes were observed in AMPKα phosphorylation (Fig 4D). In contrast, hearts stored in 1 μM dantrolene trended towards increased connexin 43 phosphorylation at S368 compared to control hearts, correlating with improved functional recovery in these hearts.

### Effects of dantrolene-supplementation during cold storage on functional recovery in older hearts

**Fig 5:**
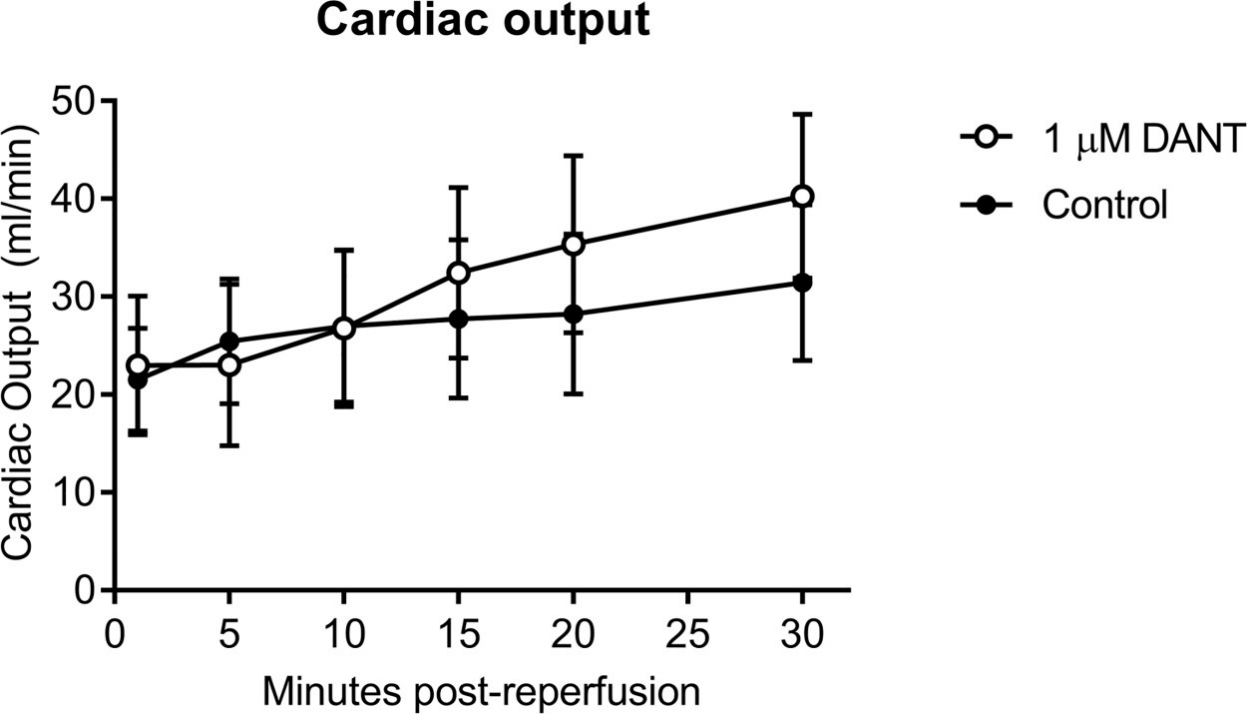
Cardiac output during 30 minutes of working heart reperfusion of 12 month old hearts after 6h cold storage in Celsior alone or supplemented with 1 μM dantrolene. Bars represent mean ± SEM; n = 7 per group.

Previous studies have shown that spontaneous Ca^2+^ release events of aged cardiomyocytes are delayed in response to dantrolene treatment however this effect is not observed in cardiomyocytes derived from young mice [24]. Since 1 μM dantrolene supplementation improved functional recovery in young hearts, we tested whether dantrolene-mediated cardiac functional recovery could be further improved after prolonged cold storage of older hearts. Despite a slight trend towards increased CO after 30 minute reperfusion, the observed recovery in 3 month old hearts was not observed in 12 month old hearts (Fig 5).

## Discussion

Previous studies have shown that dantrolene can minimise IRI in global warm ischaemia models however whether dantrolene can protect the heart after prolonged periods of cold ischaemia has not been demonstrated. The present study reports for the first time the effects of dantrolene on cardiac functional recovery in the context of prolonged donor heart cold storage. We show that supplementation of hearts with 1 μM dantrolene during hypothermic storage significantly improves aortic flow and cardiac output. In contrast, higher doses at 40 μM dantrolene were toxic and abolished cardiac recovery. The poor functional recovery observed at 40 μM dantrolene can be explained in part by myocardial necrosis and by the direct negative effect on myocardial contraction, as indicated by increased LDH release and reduced contraction bands respectively. Further, the inhibitory effect of dantrolene on Ca^2+^ release from the SR is likely to prevent reperfusion-induced hypercontracture caused by rapid SR Ca^2+^ cycling[1, 25]. Contrary to our current observations, previous reports show a dose-dependent reduction in LDH release [18]. However, a decrease in cardiac contractility has also been shown due to the muscle relaxant properties of dantrolene at comparable doses[26].

The cardioprotective signalling responsible for the functional benefits of dantrolene during ischaemia-reperfusion have not been reported. In our study, no changes were observed in AMPKα activation in dantrolene-stored hearts. Contrasting results with respect to the RISK signalling pathways were observed. Firstly, the increased ERK1/2 signalling is unlikely to explain the observed functional benefit as there was no correlation with functional recovery. Secondly, we observed a decrease in AKT phosphorylation in 1 μM dantrolene-stored hearts which contradicts with the increased AKT phosphorylation typically observed in relation to cardioprotection [27, 28]. The effect that dantrolene has on the aforementioned signalling pathways has previously not been extensively reported. However, in skeletal muscle models of caffeine-induced signalling, dantrolene does not alter AKT phosphorylation [29] but inhibits AMPKα activation [30]. Also, dantrolene inhibits ERK1/2 phosphorylation in gonadotropin-treated epithelial ovarian cancer cell lines [31]. To our knowledge, there are no reports on the effects of dantrolene on STAT3 signalling however we observed significantly increased STAT3 phosphorylation at lower concentrations of dantrolene supplementation unrelated to functional recovery. Of interest, the trend towards increased connexin 43 phosphorylation correlating with improved cardiac recovery supports the notion of a cardioprotective role for connexin 43. Despite the involvement of connexin 43 in gap junction formation and intercellular communication [32], connexin 43 has also been implicated as a mediator of cardioprotection as heterozygous connexin 43 knockout mice show loss of cardioprotection in response to ischaemic-preconditioning [33]. If connexin 43 is associated dantrolene-mediated cardioprotection, it is likely related to the ability of dantrolene to reduce intracellular calcium flux as dantrolene does not alter gap junction formation [34].

It is important to highlight that the narrow therapeutic dose range of dantrolene supplementation that corresponded to a functional benefit presents a potential limitation for clinical translation. Since dantrolene modulates calcium release from the SR, it is possible that the functional and signalling effects are calcium-dependent and can be influenced by Ca^2+^ concentrations in the perfusion system. This held true in studies where variations in the Ca^2+^ concentration of KHB buffer altered cardiac isometric force in the presence of dantrolene[35]. Another possibility is that at lower temperatures, dantrolene-mediated inhibition of calcium release is reduced as observed in studies of the RyR1 channel [8]. Further, the structural conformation of RyR2 may restrict the binding of dantrolene under normal physiological conditions [3]. However, ischaemia/reperfusion in itself significantly increases intracellular calcium stores and may provide the required modification of the RyR2 dantrolene binding site [36, 37].

It has been proposed that dantrolene can preferentially access the RyR2 binding site when the interdomain interactions are disrupted under conditions of cellular stress [6] such as with ageing. Indeed, dantrolene corrected for calcium-induced dysfunction in aged cardiomyocytes more so than in young cardiomyocytes [24] suggesting that dantrolene-mediated cardioprotection may be more prominent in hearts from older donors. In our study the cardioprotective benefit of 1 μM dantrolene-supplementation in 3-month hearts was not statistically significant for 12-month hearts. It is possible that functional recovery of older hearts (i.e., 18-month) after dantrolene-supplementation may show a larger difference compared to controls since previous work from our laboratory using older hearts showed greater cardiac recovery in 18-month versus 12-month hearts [27]. However, in our experience, functional studies utilising donor hearts 12 months and older present logistical challenges due to increased attrition and costs associated with maintaining sufficient animals to guarantee enough numbers for experimental groups. Due to the limited number of 12 month old animals available during our study, only the optimal dantrolene concentration observed from our 3 month studies was tested, however, significant changes in cardiac recovery may have been observed at different dantrolene doses.

In the current study, there are certain aspects of the experimental protocol that vary from a clinical setting. Firstly, brain death or circulatory death have not been incorporated in our model before harvesting the donor heart, which in itself can influence the extent of donor heart recovery and would represent a clinical equivalent of our experimental model. Secondly, the use of KHB (an oxygenated crystalloid buffer) for reperfusion is different from blood-based reperfusion in a transplant setting. Experimentally, this can be tested using the technically challenging heterotopic rodent heart transplant model. However, the benefit of using KHB perfusion is the absence of potentially detrimental white cells and other blood-borne products that can confound the cardiac functional effects of potential cardioprotective agents in our *ex vivo* model. Although it is possible that changing from KHB perfusion to blood-based perfusion will negate any cardioprotective benefit for dantrolene, as was observed by Preckel *et*. *al*.[17] where dantrolene showed creatine kinase reductions in a Langendorff perfusion model of IRI but no improvement in myocardial infarct size *in vivo*. Thirdly, our protocol contains heparin administration prior to harvesting the donor heart to minimise clot formation in the cardiac microvasculature that can contribute towards poor perfusion, however the legislation on clinical antemortem heparin use varies between different transplant centres.

In conclusion, we have shown that dantrolene-supplementation improves functional recovery after prolonged hypothermic storage of donor hearts from young but not older hearts. Our study demonstrates a narrow therapeutic window for dantrolene suggesting that testing in a clinically relevant model incorporating brain death may require further dose optimisation.
